# Evaluating survival in subjects with astrocytic brain tumors by dynamic susceptibility-weighted perfusion MR imaging

**DOI:** 10.1371/journal.pone.0244275

**Published:** 2021-01-06

**Authors:** Matthew L. White, Yan Zhang, Syed A. Jaffar Kazmi, Michele Aizenberg, Nicole Shonka, Fang Yu, Adams Kusi Appiah

**Affiliations:** 1 Radiology, University of Nebraska Medical Center, Omaha, Nebraska, United States of America; 2 Anatomic Pathology, Geisinger Medical Center, Danville, Pennsylvania, United States of America; 3 Neurosurgery, the University of Nebraska Medical Center, Omaha, Nebraska, United States of America; 4 Internal Medicine Division of Oncology & Hematology, University of Nebraska Medical Center, Omaha, Nebraska, United States of America; 5 Biostatistics, University of Nebraska Medical Center, Omaha, Nebraska, United States of America; Goethe University Hospital Frankfurt, GERMANY

## Abstract

**Purpose:**

Studies have evaluated the application of perfusion MR for predicting survival in patients with astrocytic brain tumors, but few of them statistically adjust their results to reflect the impact of the variability of treatment administered in the patients. Our aim was to analyze the association between the perfusion values and overall survival time, with adjustment for various clinical factors, including initial treatments and follow-up treatments.

**Materials and methods:**

This study consisted of 51 patients with astrocytic brain tumors who underwent perfusion-weighted MRI with MultiHance® at a dose of 0.1 mmol/kg prior to initial surgery. We measured the mean rCBV, the 5% & 10% maximum rCBV, and the variation of rCBV in the tumors. Comparisons were made between patients with and without 2-year survival using two-sample t-test or Wilcoxon rank-sum test for the continuous data, or chi-square and Fisher exact tests for categorical data. The multivariate cox-proportional hazard regression was fit to evaluate the association between rCBV and overall survival time, with adjustment for clinical factors.

**Results:**

Patients who survived less than 2 years after diagnosis had a higher mean and maximum rCBV and a larger variation of rCBV. After adjusting for clinical factors including therapeutic measures, we found no significant association of overall survival time within 2 years with any of these rCBV values.

**Conclusions:**

Although patients who survived less than 2 years had a higher mean and maximum rCBV and a larger variation of rCBV, rCBV itself may not be used independently for predicting 2-year survival of patients with astrocytic brain tumors.

## Introduction

The survival time of patients with astrocytic brain tumors is quite variable. For decades histological grade was the best predictor of survival [1–3]. With standard treatment, the median survival for patients with an anaplastic astrocytoma (AA) is approximately two to three years from the time of diagnosis and about one year for patients with the more aggressive glioblastoma (GBM). By contrast, patients with a low-grade astrocytoma (AST) may survive a decade or more [4,5]. However, histological tumor grade is not always consistent with the tumor prognosis. Low grade gliomas may have an aggressive course and the median survival time may be as short as three years. Some high grade gliomas behave less aggressively than expected and 3–5% of patients with GBM may survive for more than three years [4,6]. The addition of genetic and molecular classification using IDH mutation, 1p19q codeletion, MGMT promoter methylation, ATRX expression loss and epidermal growth factor receptor has improved prognostic precision [7,8]. However, due to sampling errors, histological grading, genetic classification, and molecular classification may not be accurate. There is also inter- and intrapathologist variability in pathology analysis [9]. Furthermore, neurosurgical tumor sampling carries the risks of death (0.9%), major morbidity (4%) and some deep-seated lesions are not readily accessible [9]. Given these limitations, it is a goal of neuroimaging to find non-invasively obtained indicators of survival.

Conventional anatomic MRI findings of gliomas are not always predictive of tumor grade, a non-enhancing lesion might represent a high-grade glioma rather than a low-grade glioma, and additional physiologic imaging parameters could be useful [10]. Dynamic susceptibility-weighted perfusion imaging of brain tumors can provide physiologic information about vascular endothelial proliferation, vascular density, and angiogenesis in terms of the relative CBV (rCBV) [11–14]. The degree of neovascularization in gliomas correlates with the degree of malignancy and can indicate the prognosis of these patients [15,16]. Perfusion MRI with rCBV should potentially be able to predict survival time in patients with astrocytic brain tumors. Studies conducted to analyze the relationship between rCBV and overall survival time have not demonstrated conclusive results. Some studies [17–22] have shown rCBV as a useful biomarker and others have not demonstrated rCBV as a useful biomarker for predicting survival [23–28]. Few of these studies statistically adjust their results to reflect the impact of the variability of treatment administered in the patients [25–29]. Also, there is no study taking into account the additional follow-up treatments the patients received. Treatment factors are potentially very important when analyzing survival and might greatly influence the survival analysis. We therefore retrospectively reviewed perfusion MR studies in patients with pure astrocytic brain tumors. The purpose of our study is to evaluate the association between rCBV values and patients’ 2-year overall survival times. To minimize the bias related to various clinical factors, patient age at diagnosis, sex, histopathologic grade, extent of surgery, tumor volume, and therapeutic measures were considered as potential covariates statistically. Our hypothesis was that rCBV may be used independently for predicting 2-year survival in astrocytic brain tumors.

## Materials and methods

### Patients

We searched the medical records and imaging database from our institution between March 2007 and August 2013. Fifty-one consecutive patients with pathologically proven astrocytic gliomas who underwent pretreatment MR studies, including perfusion-weighted MRI, were enrolled in this study. Entry criteria included: (1) availability of digital MR data for image processing; and (2) the presence of solid tumor components available for rCBV analysis. The exclusion criteria were: (1) evidence of systemic malignancy, metastatic disease or immune status compromise; (2) prior stereotactic biopsy; (3) death unrelated to astrocytic glioma; (4) low quality MRI due to artifact; and (5) loss to follow up within 2 years. The pathologic diagnosis was determined with specimens removed at surgical resection or stereotactic biopsy by a board-certified neuropathologist utilizing the 2007 World Health Organization Classification II–IV [30]. The interval between the preoperative MRI studies and the pathologic diagnosis was 0–36 days (mean, 4 days). We recorded patient age at diagnosis, sex, histopathologic grade, and extent of surgery. Karnofsky performance scales in a number of patients were not available so the scales were not included in the analysis.

The treatment protocols patients received until death if before 2 years or up to 2 years from diagnosis were recorded from our database. In patients treated at other institutions, we obtained the treatment information from their physicians and/or healthcare providers. In patients who died within 2 years, overall survival time was calculated as the numbers of days between the pretreatment MRI study and the date of death. Patients who died after 2 years or remained alive were considered censored on the date at the end of 2 years.

Institutional review board approval from University of Nebraska Medical Center was obtained, and informed patient consent was not required for the retrospective review of the medical records or the MR images in our database for this study. For patients who were not followed in our hospital, we contacted them or their families for confirming patients’ survival status and obtained the written informed consent from the patients who were alive. If patients were deceased consent was not required by the IRB.

### MRI technique

The MRI examinations are summarized as follows: 24 patients on Achieva (3T, Philips Medical Systems, Best, The Netherlands), 5 patients on Intera (1.5T, Philips Medical Systems, Best, The Netherlands), 18 patients on Signa HDx (3T, GE Healthcare, Milwaukee, WI), and 4 patients on Signa HDxt (1.5T, GE Healthcare, Milwaukee, WI). On all MR systems, dynamic susceptibility-weighted perfusion contrast-enhanced MR images were acquired with echo-planar imaging sequence during the first pass of a standard-dose (0.1 mmol/ kg, MultiHance Bracco, Milan, Italy) bolus at a rate of 5 mL/s. This was followed by a 20mL IV saline flush at a rate of 5 mL/s.

Perfusion imaging was conducted on the Philips MR scanners (both 1.5 and 3T) using the following parameters: TR/TE, 15-17ms/23-25ms; field of view, 220mm x 220mm; slice thickness, 3.5mm; slice gap, 3.5mm; NEX, 1; matrix, 128 x 128 x 16; time points, 60; flip angle, 7. This is PRESTO (principle of echo shifting with a train of observations; Philips Medical Systems, Best, The Netherlands) technique.

The perfusion parameters on the GE MR scanners (both 1.5 and 3T) were as follows: TR/TE, 1500-2200ms/19.6ms on 3T and 1900-2150mm/20.5 or 80ms on 1.5T; field of view, 260mm x 260mm on 3T and 300mm x 300mm on 1.5T; slice thickness, 5mm; slice gap, 5mm; NEX, 1; matrix, 128 x 128 x 16; time points, 50; flip angle, 60 on 3T and 90 on 1.5T.

The conventional anatomic MR study included the following sequences: T1-weighted, T2-weighted, FLAIR, gradient-recalled echo, and post-gadolinium axial, coronal and/or sagittal T1-weighted sequences. All patients underwent contrast enhanced high spatial resolution 3D T1-weighted imaging, and transverse images were reformatted from that data set.

### MR imaging analysis

The MR images of these patients were analyzed in conference by a neuroradiologist (M.L.Y. certified neuroradiologists for 19 years) and a radiologist (Y.Z. over 10 years of experience in neuroimaging) **with** knowledge of the diagnosis of glioma but without knowledge of the histologic grade. The radiologists reached a consensus regarding the imaging findings and ROI determination. Tumor components evaluated were designated as enhanced tumor, nonenhanced tumor, and whole tumor which included both enhanced and nonenhanced tumor.

MR images were transferred to a personal Linux workstation and processed with a series of imaging software packages, including FMRIB's Software Library (FSL) v5.0 and ImageJ, and in-house built tools [31,32]. In each case, all imaging modalities were registered to the T1-weighted post-contrast image using the brain extractions (FSL's BET tool) [33]. Perfusion data was analyzed with the ImageJ package (v1.42) [34] producing rCBV maps corrected for contrast leakage with the DSCoMAN plugin applied to the motion-corrected (FSL's mcflirt) image set [35,36]. Standard deviation (SD) maps of rCBV that reflect the variation of rCBV were also created for each tumor component with in-house built software.

On rCBV measurements, we drew ROIs as large as possible to cover a maximum of the tumor components: enhanced, nonenhanced, or whole. We primarily used the T1-weighted post-contrast image to designate the tumor components and ROI delineation. We also inspected T1-weighted, T2-weighted, FLAIR images, as well as dynamic perfusion series to verify the ROI placements and to verify that the ROIs did not include hemorrhage, blood vessels, necrosis or cystic changes. The T2/FLAIR hyperintensity surrounding the tumor, the so-called peritumoral edema, was not counted as a tumor component. The mean rCBV (rCBV_mean_) represents averaged rCBV values from all image sections that contained the tumor component.

The 5% and 10% maximum rCBV values (rCBV_5%max_ and rCBV_10%max_) of the whole tumor were computed respectively [25,37]. Additionally, two to five ROIs measuring 30–50 mm^2^ were placed on the rCBV maps in the areas with the highest visually identifiable perfusion without regard to whether the ROIs were in enhanced or non-enhanced tumor. The averaged rCBV value from these areas was chosen to represent the maximum of rCBV (rCBV_high_perfusion_) of a tumor.

**Fig 1:**
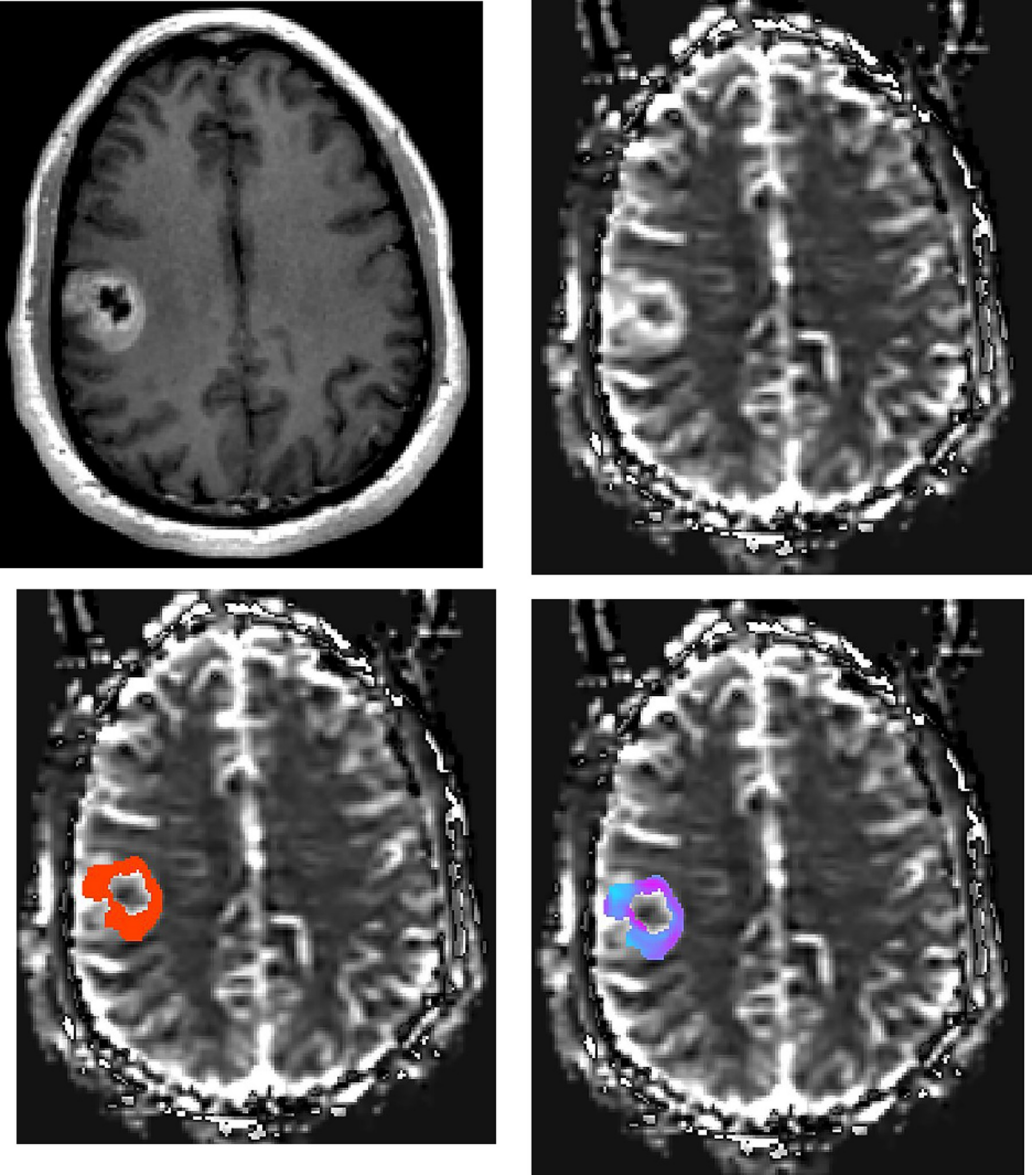
Patient with GBM. Axial (a) contrast-enhanced T1-weighted image shows the right frontal GBM. rCBV maps at the same axial level show (b) lesion with high perfusion in solid component, (c) manually drawn region of interest including contrast-enhanced tumor avoiding any cystic necrotic part to measure rCBV, and (d) standard deviation (SD) map of rCBV reflecting the variation of lesion rCBV. The variety of colors in SD map (d) represent the differences in rCBV values, where pink represents a higher value and blue represents a lower value.

The rCBV ratio was calculated by comparing the rCBV with a measurement of the contralateral normal white matter, i.e., the ipsilateral value was divided by the contralateral value. The mean and maximum values of rCBV variation (rCBV_variation_mean_ and rCBV_variation_max_), which reflect histologic heterogeneity of tumor, were calculated for each tumor component respectively via SD map of rCBV. The SD map was created utilizing a three-dimensional sphere with a radius of 3 mm centered on each voxel of the rCBV map within each tumor component (enhanced, nonenhanced and whole tumor). From the rCBV values within that sphere the SD was calculated for the central voxel. Tumor volume, which was defined as all tumor components including necrosis and cystic changes, was recorded (Fig 1).

### Statistical analysis

Comparisons were made between the patients with and without 2 year survival using two-sample t test or Wilcoxon rank sum test for the continuous data, or chi-square test and fisher exact test for categorical data when appropriate. Kaplan-Meier curve was used to describe the survival data. Cox proportional hazard regression can model association between continuous and/or categorical predictors with survival outcome with adjustment for confounding effects of various clinical factors. Therefore, two Cox proportional hazard regressions were used to assess association between the perfusion values and overall survival time, with account for confounding effects of patient’s age at diagnosis, sex, histopathologic grade, extent of surgery, tumor volume, and baseline therapeutic measures in one regression, and an additional adjustment of the receipt of any follow-up therapeutic measures in the second regression. To assess the validity or the Cox regression model, collinearity was assessed between rCBV measurements and tumor grade using regression and variance inflation factor (VIF) to assess intercorrelation and existing of collinearity between rCBV measurements and tumor grade depending on whether a large VIF value (i.e. >4) was observed [38]. In addition, supremum test was conducted to check the proportion hazard assumption of the considered Cox regression models [39]. In secondary analyses, similar Cox proportional hazard regression was conducted with restriction to patients with GBM grade. All Data analysis was performed by biostatistician (F.Y. and A.K.A) by using SAS (Version 9.4, Cary, NC).

## Results

### Patient data

Among 51 patients with astrocytic gliomas, 23 were male and 28 were female. The median age was 52.46 years, ranging from 16.91 to 84.32. Twenty patients underwent total tumor resection, 8 patients underwent subtotal resection, 1 underwent partial resection, and 22 patients underwent biopsy. The 51 astrocytic gliomas included 30 GBMs (WHO grade IV), 14 AAs (grade III), and 7 ASTs (grade II).

### Treatment

Following pathological confirmation, 35 patients (10 AA and 25 GBM) received concurrent chemoradiation therapy with temozolomide (CCRT), 1 patient (GBM) had chemotherapy alone, 10 patients (2 AST, 4 AA, and 4 GBM) underwent radiation alone, and 5 patients (AST) received no treatment. After these initial therapies, 42 patients (2 AST, 10 AA, and 30GBM) received the subsequent treatments during the time of this study as follows: adjuvant temozolomide (1 AST, 9 AA, and 23 GBM), bevacizumab (5 AA and 15 GBM), other chemotherapy agents (1 AST, 2 AA and 13 GBM), and/or radiation (3 AA and 4 GBM).

### Survival outcome

**Fig 2:**
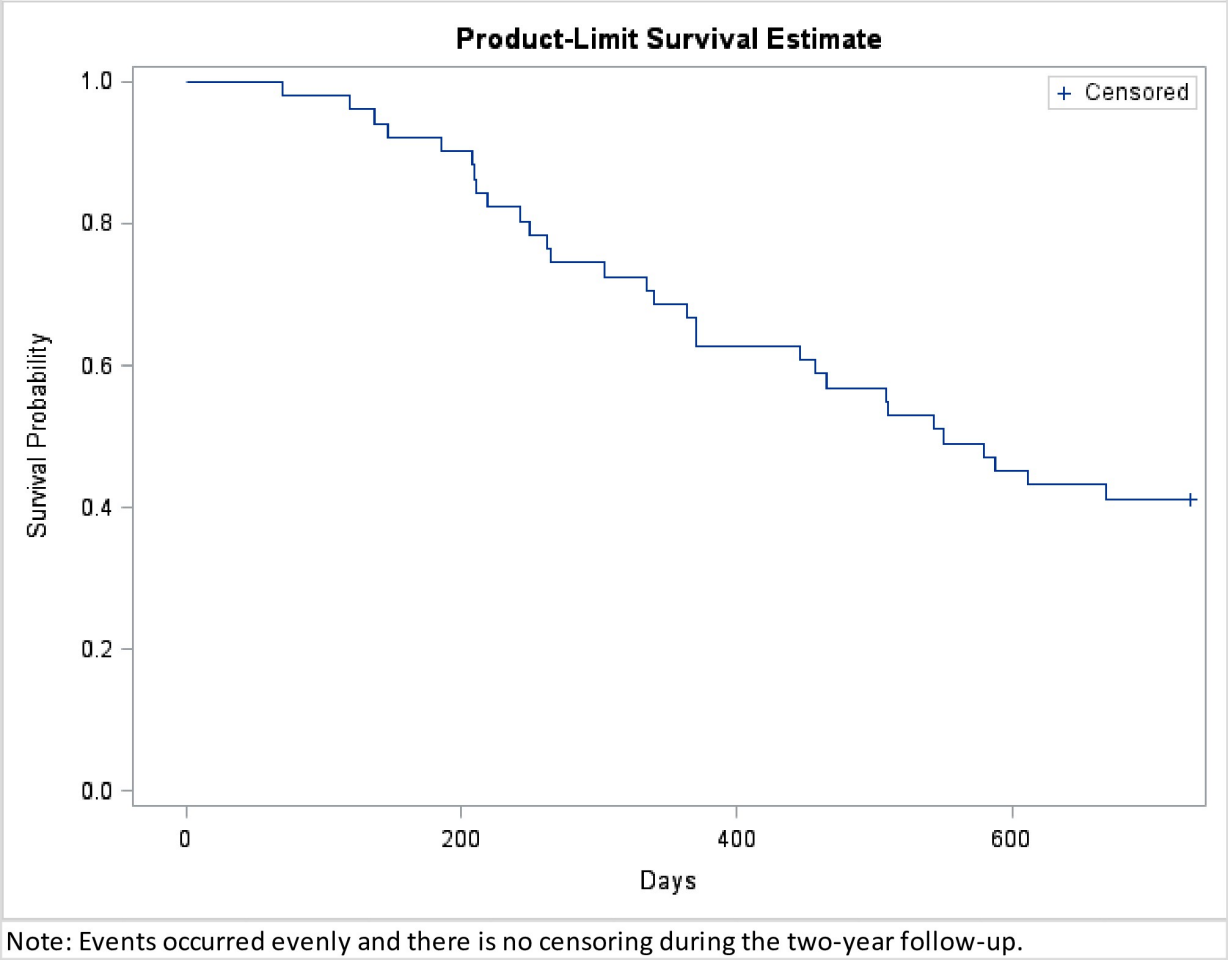
Kaplan Meier curve from MRI diagnosis to the end of 2 year follow-up.

Kaplan Meier curve was plotted to describe 2 year survival from disease diagnosis in Fig 2. Events occurred evenly and there was no censoring during the two-year follow-up. At the end of 2 years from the pretreatment MRI study, 30 out of 51 patients had died with the median overall survival time of 337 days (range, 70–669 days). The clinical factors and patients’ survival status are summarized in Table 1. Compared to the patients who survived more than 2 years, the patients who died within 2 years were older (mean 62.07 vs 43.86 y/o at diagnosis, P = 0.001) and had bigger tumor volumes (29.91 vs 9.70 cm^3^, P = 0.016). Different two year survival rates existed for patients with different pathological diagnosis (P < 0.001). Specifically, the survival rate was 100% (7 out of 7) in patients with AST, 64.29% (9 out of 14) in patients with AA, and only 16.67% (5 out of 30) in patients with GBM. The extent of surgery and sex were not significant factors related to 2-year survival.

**Table 1:** Comparison of clinical factors between patients with different statuses of 2-year survival.

	2-year survival		P value
	< 2 year (n = 30)	> 2 year (n = 21)	
**age (yr)**	62.07	43.86	0.001
**Sex**			
Female	14	14	0.158
Male	16	7	
**Pathologic Diagnosis**			
AST	0	7	<0.001*
AA	5	9	
GBM	25	5	
			
**Tumor volume (median) (cm**^**3**^**)**	29.91	9.7	0.016
**Surgical Intervention**			
Biopsy	14	8	0.210
Subtotal or partial resection	7	2	
Total resection	9	11	
**Initial Treatment**			
**AA**			
CCRT	3	7	0.580*
Chemotherapy or radiation alone	2	2	
**GBM**			
CCRT	20	5	0.556*
Chemotherapy or radiation alone	5	0	
**AA+GBM**			
CCRT	23	12	0.695*
Chemotherapy or radiation alone	7	2	
**Follow-up Treatment**			
**Adjuvant Temozolomide**			
yes	20	13	0.726
no	10	8	
**Bevacizumab**			
yes	14	6	0.193
no	16	15	
**other chemotherapy agents**			
yes	12	4	0.113
no	18	17	
**Radiation**			
yes	4	3	1.000*
no	26	18	

Note: Values are number of patients unless otherwise indicated. P values were calculated by using Chi-square test unless specified.

* Fisher-exact test was used for comparison. AST, astrocytoma; AA, anaplastic astrocytoma; GBM, glioblastoma multiforme; CCRT, concurrent chemoradiation therapy with temozolomide.

Patients receiving CCRT showed a relatively higher 2-year survival rate than those receiving chemotherapy or radiation alone in the groups with AA (7 out of 10 (70%) vs 2 out of 4 (50%), P = 0.58) and with GBM (5 out of 25 (20%) vs 0 out of 5 (0%), P = 0.556). There was no significant survival difference related to follow-up treatment between patients with and without adjuvant temozolomide, bevacizumab, other chemotherapy agents, or radiation (Table 1).

Patients who died within 2 years had a higher mean & maximum rCBV and a larger variation of rCBV than patients who survived for more than 2 years, with a statistical significance in rCBV_mean_ of nonenhanced & whole tumor, rCBV_5%max_, rCBV_10%max_, rCBV_high_perfusion_, and rCBV_variation_max_ of enhanced tumor (Table 2). The VIF values were estimated for regressions between rCBV measurements and tumor grade. The resulted VIF values ranged around 1, indicating no collinearity between rCBV measurements and tumor grade. The rCBV values were further analyzed via two Cox proportional hazard regressions with adjustment for patient age at diagnosis, sex, histopathologic grade, extent of surgery, tumor volume, and therapeutic measures. Supremum tests showed that the proportional hazard assumption was met for all considered Cox regression models. However, different from the results in Table 2, we found no significant association of overall survival time within two years with any of these rCBV values (Table 3).

**Table 2:** Comparison of perfusion values between patients with different statuses of 2-year survival.

Perfusion values	2-year survival		P value
	< 2 year (n = 30)	> 2 year (n = 21)	
**rCBV**_**mean**_ **of nonenhanced tumor (ratio)**	2.55 +/- 1.07	1.69 +/- 0.72	0.01
**rCBV**_**mean**_ **of enhanced tumor (ratio)**	3.78 [3.09–4.7]	3 [1.4–4.75]	0.206
**rCBV**_**mean**_ **of whole tumor (ratio)**	3.54 [2.13–4.24]	1.87 [1.46–2.8]	0.013
**rCBV**_**5%max**_ **(ratio)**	9.18 [6.36–11.22]	4.75 [3.99–7.74]	0.002
**rCBV**_**10%max**_ **(ratio)**	7.78 [5.96–9.91]	4.1 [3.51–6.64]	0.002
**rCBV**_**high_perfusion**_ **(ratio)**	6.75 [4.73–10.39]	2.7 [2.1–3.94]	<0.001
**rCBV**_**variation_mean**_ **of nonenhanced tumor**	13.55 +/- 7.82	11.21 +/- 5.93	0.344
**rCBV**_**variation_mean**_ **of enhanced tumor**	17.22 [12.17–22.95]	7.32 [6.16–10.69]	0.109
**rCBV**_**variation_mean**_ **of whole tumor**	14.59 +/- 9.01	11.97 +/- 7.38	0.59
**rCBV**_**variation_max**_ **of nonenhanced tumor**	41.27 [29.32–72.04]	41.95 [30.97–62.42]	0.917
**rCBV**_**variation_max**_ **of enhanced tumor**	59.38 +/- 32.60	32.58 +/- 18.51	0.026
**rCBV**_**variation_max**_ **of whole tumor**	55.35 [34.32–79.94]	42.77 [34.08–57.85]	0.227

Note: values are the mean +/- standard deviation or the median and [first quartile—third quartile]. rCBV_mean_, mean rCBV; rCBV_5%max_, 5% maximum rCBV; rCBV_10%max_, 10% maximum rCBV; rCBV_high_perfusion_, maximum rCBV; rCBV_variation_mean_, mean rCBV variation; rCBV_variation_max_, maximum rCBV variation.

**Table 3:** Association between perfusion values and overall survival times *.

Perfusion Values	Hazard Ratio	Wald Lower	Wald Upper	P value
**rCBV**_**mean**_ **of nonenhanced tumor (ratio)**	1.699	0.754	3.829	0.20
**rCBV**_**mean**_ **of enhanced tumor (ratio)**	0.994	0.805	1.229	0.96
**rCBV**_**mean**_ **of whole tumor (ratio)**	0.994	0.809	1.220	0.95
**rCBV**_**5%max**_ **(ratio)**	1.076	0.971	1.193	0.16
**rCBV**_**10%max**_ **(ratio)**	1.079	0.959	1.214	0.21
**rCBV**_**high_perfusion**_ **(ratio)**	1.010	0.910	1.120	0.85
**rCBV**_**variation_mean**_ **of nonenhanced tumor**	0.999	0.994	1.005	0.81
**rCBV**_**variation_mean**_ **of enhanced tumor**	0.999	0.991	1.006	0.77
**rCBV**_**variation_mean**_ **of whole tumor**	0.994	0.988	1.001	0.09
**rCBV**_**variation_max**_ **of nonenhanced tumor**	1.000	0.998	1.002	0.75
**rCBV**_**variation_max**_ **of enhanced tumor**	0.999	0.998	1.001	0.41
**rCBV**_**variation_max**_ **of whole tumor**	1.000	0.999	1.002	0.64

Note: * Association are calculated via Cox proportional hazard regression with adjustment of patient age at diagnosis, sex, histopathologic grade, extent of surgery, tumor volume, and therapeutic measures. rCBV_mean_, mean rCBV; rCBV_5%max_, 5% maximum rCBV; rCBV_10%max_, 10% maximum rCBV; rCBV_high_perfusion_, maximum rCBV; rCBV_variation_mean_, mean rCBV variation; rCBV_variation_max_, maximum rCBV variation.

## Discussion

In our study, patients who died within 2 years had a higher rCBV in tumor than patients who survived more than 2 years. This difference was more apparent in the maximum rCBV (i.e. rCBV_5%max_, rCBV_10%max_, and rCBV_high_perfusion_) (Table 2). Using higher percentage values of rCBV to evaluate the maximum rCBV of a tumor has been reported in previous studies [25,37]. This methodology minimizes the effect of possible outlier values encountered when choosing a single pixel ROI or ROI with only a few pixels [19] to calculate the maximum CBV value across all tumor sections. Another approach for measuring maximum rCBV in previous studies is to place an ROI in the area with the highest visually identifiable perfusion [19,23]. However, due to the limitations of visual recognition, it is not always easy to select the single best ROI from all tumor sections, especially when there are multiple regions showing similar elevated perfusion. In our study, we averaged rCBV values from several (2–5) ROIs for evaluating the maximum rCBV to avoid outlying rCBV values affecting the data. Of course, due to the averaging effect, the method we used will to some extent lower these values and result in a lower maximum rCBV (rCBV_high_perfusion_). We noticed that the average rCBV_high_perfusion_ we measured was less than the average of rCBV_5%max_ and rCBV_10%max_ (Table 2).

Unlike our results, in M Law et al.’s study [23], which consisted of 152 low- and high-grade astrocytic brain tumors, the maximum of rCBV was not significantly associated with survival. They used the highest single value recorded from regions of highest perfusion as the maximum rCBV value. This method increases the risk that the recorded value is higher than the true maximum value due to the possible existence of a small vessel in the measurement. S.J. Mills et al. [24], who performed a survival analysis of 27 patients with low- and high-grade gliomas, also claimed that CBV provides no prognostic information. However, unlike how we measured rCBV from all the image sections that contained the tumor components, they drew ROIs only on 3 sections through enhancing tumor components, excluding nonenhancing areas. Restricting ROI analysis to enhancing tumor may cause a significant bias because the maximum CBV might not be detected within the enhancing tumor component [29]. Also, using only 3 sections would limit sampling for measurement. In other studies, which found no significant relationship between survival rates and mean or maximum rCBV values in high-grade gliomas, the measurement of tumor rCBV included tumor necrosis [25,26]. This may cause the measured values to be inaccurate.

As with our results, some studies [17–22] have also reported that rCBV values are associated with overall survival time. However, few of these studies evaluated the predictive role of treatment [29]. It has been confirmed that CCRT followed by adjuvant temozolomide improved the survival in patients with high grade glioma [40]. Our data also showed that this treatment was correlated with a relatively better 2 year survival (Table 1).

Our patients also received Bevacizumab (n = 20), other chemotherapy agents (n = 16), and additional radiation (n = 7) during the follow-up after initial treatment. No improvement of the 2 year survival was found in these patients (Table 1).

It has been reported that the variation of fractional anisotropy values on DTI is useful for differentiating low- and high-grade gliomas [41]. To the best of our knowledge, however, there is no study relating rCBV variation to tumor grade and patients’ survival. In our study, we created SD maps of rCBV and calculated rCBV variation. Patients who died within 2 years had greater variation of rCBV values in tumor than those who survived for more than 2 years, strongest effect noted with the maximum value of rCBV variation of the enhanced tumor (59.38 vs 32.58) (Table 2). Compared with low-grade gliomas, increased focal vascularity can be found in high-grade gliomas. Also, some low-grade gliomas dedifferentiate into more malignant forms with time [42–45] and potentially related to this process is that high-grade tumors can present as malignant foci within an otherwise benign-appearing mass. Therefore, high-grade gliomas are histologically more heterogeneous. This feature is even without considering macroscopic tumor cystic change, necrosis, and hemorrhage. We speculate that the tumor components with maximum rCBV variation might reflect regional heterogeneity of histopathology and have more malignant foci.

This study analyzed all tumors, low-grade and high-grade, as a whole. This is because histological tumor grade is not always consistent with the tumor progression and conventional anatomic MRI appearances of gliomas can be misleading. There have been reports that patients with GBM may survive for a longer period of time while patients with low-grade glioma may have a relatively short survival time [4,6]. Moreover, the accuracy of pathological diagnosis may be affected by sampling. To confirm whether CBV can be used as an independent factor to predict the survival time of patients, we adjusted the confounding effects of pathologic grade when assessing the results.

In this study, we also performed a separate analysis of the CBV in the GBM only group. Between the GBM patients with different statuses of 2-year survival, we did not see any significant differences in clinical factors or perfusion values (Tables 4 and 5). As for the association between perfusion values and overall survival times in GBM patients, we obtained similar magnitude of hazard ratio and statistical significance to the analysis of all data including both low- and high-grade tumors (Table 6).

**Table 4:** Comparison of clinical factors between GBM patients with different statuses of 2-year survival.

	2-year survival		P value
	< 2 year (n = 25)	> 2 year (n = 5)	
**age (yr)**	59.19	59.15	0.697
**Sex**			
Female	11	3	0.642
Male	14	2	
			
**Tumor volume (median) (cm**^**3**^**)**	36.71	15.92	0.075
**Surgical Intervention**			
Biopsy	9	0	0.061
Subtotal or partial resection	7	0	
Total resection	9	5	
**Initial Treatment**			
CCRT	20	5	0.566
Chemotherapy or radiation alone	5	0	
**Follow-up Treatment**			
**Adjuvant Temozolomide**			
yes	18	5	0.304
no	7	0	
**Bevacizumab**			
yes	12	3	1.000
no	13	2	
**other chemotherapy agents**			
yes	11	2	1.000
no	14	3	
**Radiation**			
yes	4	0	1.000
no	21	5	

Note: Values are number of patients unless otherwise indicated. P values were calculated by using non-parametric Wilcoxon rank sum test on continuous data and Fisher exact test on categorical data. GBM, glioblastoma multiforme; CCRT, concurrent chemoradiation therapy with temozolomide.

**Table 5:** Comparison of perfusion values between GBM patients with different statuses of 2-year survival.

Perfusion values	2-year survival		P value
	< 2 year (n = 25)	> 2 year (n = 5)	
**rCBV**_**mean**_ **of nonenhanced tumor (ratio)**	2.70 +/- 1.15	1.32 +/- 1.87	0.152
**rCBV**_**mean**_ **of enhanced tumor (ratio)**	3.90 [3.15–4.70]	4.75 [3.35–5.19]	0.675
**rCBV**_**mean**_ **of whole tumor (ratio)**	3.63 [2.70–4.39]	4.75 [3.35–4.76]	0.436
**rCBV**_**5%max**_ **(ratio)**	9.44 [6.83–11.22]	10.20 [9.61–10.31]	0.911
**rCBV**_**10%max**_ **(ratio)**	7.91 [6.27–9.91]	9.31 [8.12–9.41]	1.000
**rCBV**_**high_perfusion**_ **(ratio)**	7.11 [5.55–10.39]	6.32 [6.04–7.42]	0.578
**rCBV**_**variation_mean**_ **of nonenhanced tumor**	13.02 [8.58–22.83]	12.65 [12.65–12.65]	1.000
**rCBV**_**variation_mean**_ **of enhanced tumor**	17.24 [12.17–22.95]	8.82 [7.32–24.34]	0.675
**rCBV**_**variation_mean**_ **of whole tumor**	18.89 [8.52–20.76]	22.37 [22.37–22.37]	0.335
**rCBV**_**variation_max**_ **of nonenhanced tumor**	46.87 [24.09–72.13]	22.45 [22.45–22.45]	0.386
**rCBV**_**variation_max**_ **of enhanced tumor**	63.35 +/- 32.30	42.62 +/- 16.83	0.179
**rCBV**_**variation_max**_ **of whole tumor**	61.76 +/- 32.69	42.62 +/- 16.83	0.217

Note: values are the mean +/- standard deviation or the median and [first quartile—third quartile]. P values were calculated by using two sample t test or non-parametric Wilcoxon rank sum test when normality assmption was not met. rCBV_mean_, mean rCBV; rCBV_5%max_, 5% maximum rCBV; rCBV_10%max_, 10% maximum rCBV; rCBV_high_perfusion_, maximum rCBV; rCBV_variation_mean_, mean rCBV variation; rCBV_variation_max_, maximum rCBV variation.

**Table 6:** Association between perfusion values and overall survival times in GBM patients*.

Perfusion Values	Hazard Ratio	Wald Lower	Wald Upper	P value
**rCBV**_**mean**_ **of nonenhanced tumor (ratio)**	2.373	0.603	9.343	0.22
**rCBV**_**mean**_ **of enhanced tumor (ratio)**	0.943	0.736	1.209	0.65
**rCBV**_**mean**_ **of whole tumor (ratio)**	1.004	0.801	1.257	0.98
**rCBV**_**5%max**_ **(ratio)**	1.082	0.950	1.233	0.23
**rCBV**_**10%max**_ **(ratio)**	1.077	0.927	1.253	0.33
**rCBV**_**high_perfusion**_ **(ratio)**	1.010	0.910	1.120	0.85
**rCBV**_**variation_mean**_ **of nonenhanced tumor**	0.997	0.991	1.003	0.37
**rCBV**_**variation_mean**_ **of enhanced tumor**	0.996	0.986	1.006	0.40
**rCBV**_**variation_mean**_ **of whole tumor**	0.992	0.984	1.001	0.07
**rCBV**_**variation_max**_ **of nonenhanced tumor**	1.000	0.998	1.002	0.82
**rCBV**_**variation_max**_ **of enhanced tumor**	1.000	0.998	1.001	0.75
**rCBV**_**variation_max**_ **of whole tumor**	1.001	0.999	1.002	0.57

Note: * Association are calculated via Cox proportional hazard regression with adjustment of patient age at diagnosis, sex, histopathologic grade, extent of surgery, tumor volume, and therapeutic measures. rCBV_mean_, mean rCBV; rCBV_5%max_, 5% maximum rCBV; rCBV_10%max_, 10% maximum rCBV; rCBV_high_perfusion_, maximum rCBV; rCBV_variation_mean_, mean rCBV variation; rCBV_variation_max_, maximum rCBV variation.

The results of this study do not support our hypothesis. There was no significant association of 2-year survival with rCBV when the data were further analyzed with adjustment for clinical factors. Theoretically, rCBV that reflects the degree of neovascularization is related to the malignancy of the tumor and should in turn greatly affects the survival of patients. However, there are many factors that can cause inaccurate measurements of rCBV, such as the presence of small blood vessels and cystic changes in the tumor. Although rCBV was not an independent predictor of survival time, when we simply compared tumor rCBV without adjusting for clinical factors, patients who die within two years did have a higher rCBV and a larger variation of rCBV than patients who survived for more than two years. Therefore, clinicians can make a preliminary prediction of the patient's prognosis to some extent based on the rCBV of the tumor before surgery and subsequent treatment. The analysis of rCBV still has important clinical value.

There were limitations to our study. We evaluated a moderate number of patients in a retrospective manner. We were not able to control the application of therapeutic radiation dose, cycles of adjuvant temozolomide, and types of additional chemotherapeutic agents utilized for follow-up treatments but we did adjust our statistical analysis to remove any confounding treatment effects. Our study did not evaluate genetic and molecular features that could affect survival. However, previous work has demonstrated that rCBV measurements could be used to predict patient overall survival independent of the molecular subclasses of GBM [22,46]. There is potentially some limitation on the analysis due to not all examinations being performed on the same scanner with the same technique since this is a retrospective analysis. This was in part overcome by processing the data using the same software and normalizing the rCBV values with CBV measurements from the opposite hemisphere. The MRI sequence perfusion parameters varied from recent ASFNR perfusion guidelines but these guidelines were published after the patient data was collected [47]. In addition, the ASFNR guidelines do not apply to the Philips MRI PRESTO technique [47]. Each of our cases was judged to have a good contrast bolus detected by source image analysis. The visualization of the contrast bolus was likely optimized by the utilization of Multihance, which is a high relaxivity contrast agent.

## Conclusion

The association between rCBV and 2-year overall survival times in patients with pure astrocytic brain tumors were analyzed, adjusting for clinical variables. The additional follow-up treatments were evaluated for the first time in conjunction with rCBV to assess the impact on the survival. Although, patients who survived less than 2 years had a higher mean and maximum rCBV and a larger variation of rCBV, rCBV itself may not be used independently for predicting the 2 year survival of these patients.

## Supporting information

S1 Table (XLSX)

## References

[1] Burger PC. Malignant astrocytic neoplasms: classification, pathologic anatomy, and response to treatment. Semin Oncol 1986;13:16–26. 3006257

[2] Bauman G, Lote K, Larson D, Stalpers L, Leighton C, Fisher B, et al Pretreatment factors predict overall survival for patients with low-grade glioma: a recursive partitioning analysis. Int J Radiat Oncol Biol Phys 1999;45:923–929. 10.1016/s0360-3016(99)00284-9 10571199

[3] Scott CB, Scarantino C, Urtasun R, Movsas B, Jones CU, Simpson JR, et al Validation and predictive power of Radiation Therapy Oncology Group (RTOG) recursive partitioning analysis classes for malignant glioma patients: a report using RTOG 90–06. Int J Radiat Oncol Biol Phys 1998;40:51–55. 10.1016/s0360-3016(97)00485-9 9422557

[4] Grier JT, Batchelor T. Low-grade gliomas in adults. Oncologist 2006;11:681–693. 10.1634/theoncologist.11-6-681 16794247

[5] Behin A, Hoang-Xuan K, Carpentier AF, Delattre JY. Primary brain tumours in adults. Lancet 2003;25:323–331. 10.1016/S0140-6736(03)12328-8 12559880

[6] Krex D, Klink B, Hartmann C, von Deimling A, Pietsch T, Simon M, et al Long-term survival with glioblastoma multiforme. Brain 2007;130:2596–2606. 10.1093/brain/awm204 17785346

[7] Leeper HE, Caron AA, Decker PA, Jenkins RB, Lachance DH, Giannini C. IDH mutation, 1p19q codeletion and ATRX loss in WHO grade II gliomas. Oncotarget 2015;6:30295–30305. 10.18632/oncotarget.4497 26210286 PMC4745799

[8] Montano N, Cenci T, Martini M, D'Alessandris QG, Pelacchi F, Ricci-Vitiani L, et al Expression of EGFRvIII in Glioblastoma: Prognostic Significance Revisited. Neoplasia 2011; 13: 1113–1121. 10.1593/neo.111338 22241957 PMC3257186

[9] Jackson RJ, Fuller GN, Abi-Said D, Lang FF, Gokaslan ZL, Shi WM, et al Limitations of stereotactic biopsy in the initial management of gliomas. Neuro Oncol 2001;3:193–200. 10.1093/neuonc/3.3.193 11465400 PMC1920616

[10] Scott JN, Brasher PM, Sevick RJ, Rewcastle NB, Forsyth PA. How often are nonenhancing supratentorial gliomas malignant? A population study. Neurology 2002;59(6):947–949. 10.1212/wnl.59.6.947 12297589

[11] Aronen HJ, Gazit IE, Louis DN, Buchbinder BR, Pardo FS, Weisskoff RM, et al Cerebral blood volume maps of gliomas: comparison with tumor grade and histologic findings. Radiology 1994;191:41–51. 10.1148/radiology.191.1.8134596 8134596

[12] Sugahara T, Korogi Y, Kochi M, Ikushima I, Hirai T, Okuda T, et al Correlation of MR imaging-determined cerebral blood volume maps with histologic and angiographic determination of vascularity in gliomas. AJR Am J Roentgenol 1998;171:1479–1486. 10.2214/ajr.171.6.9843274 9843274

[13] Knopp EA, Cha S, Johnson G, Mazumdar A, Golfinos JG, Zagzag D, et al Glial neoplasms: dynamic contrast-enhanced T2*-weighted MR imaging. Radiology 1999;211:791–798. 10.1148/radiology.211.3.r99jn46791 10352608

[14] Wong JC, Provenzale JM, Petrella JR. Perfusion MR imaging of brain neoplasms. AJR Am J Roentgenol 2000;174:1147–1157. 10.2214/ajr.174.4.1741147 10749268

[15] Lopes MB. Angiogenesis in brain tumors. Microsc Res Tech 2003;60:225–230. 10.1002/jemt.10260 12539176

[16] Leon SP, Folkerth RD, Black PM. Microvessel density is a prognostic indicator for patients with astroglial brain tumors. Cancer 1996;77:362–372. 10.1002/(SICI)1097-0142(19960115)77:2<362::AID-CNCR20>3.0.CO;2-Z 8625246

[17] Hirai T, Murakami R, Nakamura H, Kitajima M, Fukuoka H, Sasao A, et al Prognostic value of perfusion MR imaging of high-grade astrocytomas: long-term follow-up study. AJNR Am J Neuroradiol 2008;29:1505–1510. 10.3174/ajnr.A1121 18556364 PMC8119049

[18] Akgoz A, Rahman R, You H, Qu J, Hamdan A, Seethamraju RT, et al Spin-echo echo-planar perfusion prior to chemoradiation is a strong independent predictor of progression-free and overall survival in newly diagnosed glioblastoma. J Neurooncol 2014;119:111–119. 10.1007/s11060-014-1454-x 24792644

[19] Bisdas S, Kirkpatrick M, Giglio P, Welsh C, Spampinato MV, Rumboldt Z. Cerebral Blood Volume Measurements by Perfusion-Weighted MR Imaging in Gliomas: Ready for Prime Time in Predicting Short-Term Outcome and Recurrent Disease? AJNR Am J Neuroradiol 2009;30:681–688. 10.3174/ajnr.A1465 19179427 PMC7051766

[20] Çoban G, Mohan S, Kural F, Wang S, O'Rourke DM, Poptani H. Prognostic Value of Dynamic Susceptibility Contrast-Enhanced and Diffusion-Weighted MR Imaging in Patients with Glioblastomas. AJNR Am J Neuroradiol 2015;36:1247–1252. 10.3174/ajnr.A4284 25836728 PMC7965272

[21] Bonekamp D, Deike K, Wiestler B, Wick W, Bendszus M, Radbruch A, et al Association of overall survival in patients with newly diagnosed glioblastoma with contrast-enhanced perfusion MRI: Comparison of intraindividually matched T1—and T2 (*) -based bolus techniques. J Magn Reson Imaging 2015;42:87–96. 10.1002/jmri.24756 25244574

[22] Jain R, Poisson L, Narang J, Gutman D, Scarpace L, Hwang SN, et al Genomic mapping and survival prediction in glioblastoma: molecular subclassification strengthened by hemodynamic imaging biomarkers. Radiology 2013;267:212–220. 10.1148/radiol.12120846 23238158 PMC3606543

[23] Law M, Young RJ, Babb JS, Peccerelli N, Chheang S, Gruber ML, et al Gliomas: predicting time to progression or survival with cerebral blood volume measurements at dynamic susceptibility-weighted contrast-enhanced perfusion MR imaging. Radiology 2008;247:490–498. 10.1148/radiol.2472070898 18349315 PMC3774106

[24] Mills SJ, Patankar TA, Haroon HA, Balériaux D, Swindell R, Jackson A. Do cerebral blood volume and contrast transfer coefficient predict prognosis in human glioma? AJNR Am J Neuroradiol 2006;27:853–858. 16611778 PMC8133992

[25] Sanz-Requena R, Revert-Ventura A, Martí-Bonmatí L, Alberich-Bayarri A, García-Martí G. Quantitative MR perfusion parameters related to survival time in high-grade gliomas. Eur Radiol 2013;23:3456–3465. 10.1007/s00330-013-2967-y 23839170

[26] Zacharaki EI, Morita N, Bhatt P, O'Rourke DM, Melhem ER, Davatzikos C. Survival analysis of patients with high-grade gliomas based on data mining of imaging variables. AJNR Am J Neuroradiol 2012;33:1065–1071. 10.3174/ajnr.A2939 22322603 PMC4373623

[27] Crawford FW, Khayal IS, McGue C, Saraswathy S, Pirzkall A, Cha S, et al Relationship of pre-surgery metabolic and physiological MR imaging parameters to survival for patients with untreated GBM. J Neurooncol 2009;91:337–351. 10.1007/s11060-008-9719-x 19009235 PMC3022444

[28] Paik W, Kim HS, Choi CG, Kim SJ. Pre-Operative Perfusion Skewness and Kurtosis Are Potential Predictors of Progression-Free Survival after Partial Resection of Newly Diagnosed Glioblastoma. Korean J Radiol 2016;17:117–126. 10.3348/kjr.2016.17.1.117 26798224 PMC4720799

[29] Akgoz A, Rahman R, You H, Qu J, Hamdan A, Seethamraju RT, et al Spin-echo echo-planar perfusion prior to chemoradiation is a strong independent predictor of progression-free and overall survival in newly diagnosed glioblastoma. J Neurooncol 2014;119:111–119. 10.1007/s11060-014-1454-x 24792644

[30] Louis DN, Ohgaki H, Wiestler OD, Cavenee WK, Burger PC, Jouvet A, et al The 2007 WHO classification of tumours of the central nervous system. Acta Neuropathol 2007;114:97–109. 10.1007/s00401-007-0243-4 17618441 PMC1929165

[31] Smith SM, Jenkinson M, Woolrich MW, Beckmann CF, Behrens TE, Johansen-Berg H, et al Advances in functional and structural MR image analysis and implementation as FSL. Neuroimage 2004;23 Suppl1:S208–219. 10.1016/j.neuroimage.2004.07.051 15501092

[32] Jenkinson M, Beckmann CF, Behrens TE, Woolrich MW, Smith SM. FSL. Neuroimage 2012;62:782–790. 10.1016/j.neuroimage.2011.09.015 21979382

[33] Smith SM. Fast robust automated brain extraction. Hum Brain Mapp 2002;17:143–155. 10.1002/hbm.10062 12391568 PMC6871816

[34] Rasband WS. ImageJ, U.S. National Institutes of Health, Bethesda, Maryland, USA, imagej.nih.gov/ij/, 1997–2016.

[35] Boxerman JL, Schmainda KM, Weisskoff RM. Relative cerebral blood volume maps corrected for contrast agent extravasation significantly correlate with glioma tumor grade, whereas uncorrected maps do not. AJNR Am J Neuroradiol 2006;27: 859–867. 16611779 PMC8134002

[36] Jenkinson M, Bannister P, Brady M, Smith S. Improved optimization for the robust and accurate linear registration and motion correction of brain images. Neuroimage 2002;17:825–841. 10.1016/s1053-8119(02)91132-8 12377157

[37] Law M, Young R, Babb J, Pollack E, Johnson G. Histogram analysis versus region of interest analysis of dynamic susceptibility contrast perfusion MR imaging data in the grading of cerebral gliomas. AJNR Am J Neuroradiol 2007; 28:761–766. 17416835 PMC7977348

[38] Smith T, Smith B. Graphing the probability of event as a function of time using survivor function estimates and the SAS® System's PROC PHREG. SAS Conference Proceedings: Western Users of SAS Software 2005. September 21–23, 2005, San Jose, California. https://lexjansen.com/wuss/2005/hands_on_workshops/how_graphing_the_probability.pdf.

[39] Hiller L, Marshall A, Dunn J. Assessing violations of the proportional hazards assumption in Cox regression: does the chosen method matter? Trials 2015;16(Suppl 2):P134.

[40] Byung Sup Kim Ho Jun Seol, Nam Do-Hyun, Park Chul-Kee, Il Han Kim Tae Min Kim, et al Concurrent Chemoradiotherapy with Temozolomide Followed by Adjuvant Temozolomide for Newly Diagnosed Glioblastoma Patients: A Retrospective Multicenter Observation Study in Korea. Cancer Res Treat. 2017;49: 193–203. 10.4143/crt.2015.473 27384161 PMC5266397

[41] White ML, Zhang Y, Yu F, Jaffar Kazmi SA. Diffusion tensor MR imaging of cerebral gliomas: evaluating fractional anisotropy characteristics. AJNR Am J Neuroradiol 2011;32:374–381. 10.3174/ajnr.A2267 20947645 PMC7965729

[42] Louis DN, Reifenberger G, Brat DJ, Ellison DW. Tumors: introduction and neuroepithelial tumors In: Love S, Louis DN, Ellison DW, eds. Greenfield’s Neuropathology. 8th ed. London, UK: Hodder Arnold; 2008:1821–1936.

[43] Jayaraman MV, Boxerman JL. Adult brain tumors In: Atlas SW, ed. Magnetic Resonance Imaging of the Brain and Spine. 4th ed. Philadelphia: Lippincott Williams & Wilkins; 2009:445–504.

[44] VandenBerg SR. Current diagnostic concepts of astrocytic tumors. J Neuropathol Exp Neurol 1992;51:644–657. 10.1097/00005072-199211000-00008 1362439

[45] Nafe R, Van de Nes J, Yan B, Schlote W. Distribution of nuclear size and internuclear distance are important criteria for grading astrocytomas. Clin Neuropathol 2006;25:48–56. 16465775

[46] Jain R, Poisson L, Narang J, Gutman D, Scarpace L, Hwang SN, et al Genomic mapping and survival prediction in glioblastoma: molecular subclassification strengthened by hemodynamic imaging biomarkers. Radiology 2013;267:212–220. 10.1148/radiol.12120846 23238158 PMC3606543

[47] Welker K, Boxerman J, Kalnin A, Kaufmann T, Shiroishi M, Wintermark M. ASFNR recommendations for clinical performance of MR dynamic susceptibility contrast perfusion imaging of the brain. AJNR Am J Neuroradiol 2015;36:E41–51. 10.3174/ajnr.A4341 25907520 PMC5074767

